# Sebaceous adenitis in Swedish dogs, a retrospective study of 104 cases

**DOI:** 10.1186/1751-0147-50-11

**Published:** 2008-05-25

**Authors:** Elisabeth Hernblad Tevell, Kerstin Bergvall, Agneta Egenvall

**Affiliations:** 1 Bagarmossen Small Animal Hospital, Ljusnevagen 17, SE-128 48 Bagarmossen, Sweden; 2 Department of Clinical Sciences, Division of Small Animal Clinical Sciences, Swedish University of Agriculture, Box 7054, SE-750 07 Uppsala, Sweden; 3 Department of Clinical Sciences, Division of Ruminant Medicine and Epidemiology, Swedish University of Agriculture, Box 7054, SE-750 07 Uppsala, Sweden

## Abstract

**Background:**

Sebaceous adenitis (SA) is an uncommon, immune mediated skin disease in dogs. The aim was to retrospectively investigate SA in dogs in Sweden with respect to breed, sex and age distribution. A second aim was to retrospectively compare clinical signs in dogs with generalized SA and to estimate the survival after diagnosis in the English springer spaniel, standard poodle and the akita.

**Methods:**

In total 34 Swedish veterinarians contributed with 104 clinically and histologically verified SA cases. Breed, gender and age at diagnosis were registered for each case. The degree of clinical signs at time for diagnosis and at follow-up and information about treatments, concurrent diseases and euthanasia were recorded for the springer spaniels, standard poodles and akitas using a standardized questionnaire.

**Results:**

A total of 104 cases of SA were included; most cases were recorded for the springer spaniel (n = 25), standard poodle (n = 21) and the akita (n = 10). These three breeds, together with the lhasa apso and the chow-chow, were the most common when national registry data from the Swedish Board of Agriculture and Swedish Kennel Club were considered. The mean age at diagnosis was 4.8 years. The proportion of males was 61%. When the springer spaniels, standard poodles and the akitas with generalized signs were compared (n = 51), the spaniels showed significantly more severe clinical signs than the poodles at diagnosis regarding alopecia, seborrhoea, pyoderma and the overall severity of clinical signs. At follow-up, the degree of clinical signs for otitis externa and pyoderma differed significantly between the breeds. The estimated median survival time was 42 months.

In dogs where data regarding survival was available at the end of the study (n = 44), SA was reported to be the reason for euthanasia in 14 dogs, whereof 7 within 24 months after diagnosis.

**Conclusion:**

The result of this study implicates that the English springer spaniel is a breed predisposed to SA and that it has more severe clinical signs than the standard poodle. A large proportion of the dogs (spaniel, poodle and akita) investigated regarding survival were reported to have been euthanized to great extent due to the disease.

## Background

Sebaceous adenitis (SA) is an uncommon, immune mediated skin disease in dogs. In other species, e.g. the cat, rabbit, horse and man, it is rarely reported [1-7].

The canine disease was first described by Scott in 1986 [8] and then by Rosser and Dunstan [9-11]. The condition has been reported in over 50 breeds [1,8-14] and the disease is well documented in the standard poodle [9,11] and the akita [12]. Breed predilection is further proposed in the vizla, samoyed and possibly also the chow-chow [1,13,14].

Histologically, SA is associated with a granulomatous immunologic reaction, ultimately destroying the sebaceous glands [8-12]. The disease can be localized or generalized and some forms of SA have only transient inflammatory reactions and mild clinical signs [10,11].

Clinical signs include varying degree of alopecia, hyperkeratosis and seborrhoea with follicular casts as a distinctive feature, especially in the chronic phase of the disease. Lesions commonly start dorsally on the head, neck and pinnae and proceed caudally and later involve the trunk, sparing the ventral abdomen and often also the extremities. It has been suggested that type of hair-coat influences clinical presentation, with short-haired dogs mainly developing multifocal erythematous papules or plaques that progress to annular alopecic scaling lesions, that can later coalesce. Long-haired dogs mainly show diffuse hyperkeratosis, seborrhoea and follicular casts, whereas in some references the clinical description is divided even further between breeds [13-15]. Pruritus is usually not an initial feature, but can develop in case of secondary pyoderma [1,9-16]. Treatment in mild cases of SA is often symptomatic and topical, based on shampoos, humectants and oil packings. Oral fatty acid supplementation is often recommended [15]. In more severe cases systemic retinoids [15,16] or corticosteroids [14,15] have been used, as has oral vitamin A (retinol) [14]. Lately, treatment with cyclosporine has shown to be beneficial [17,18]. Secondary pyoderma and otitis externa is treated when appropriate.

The prognosis depends on the severity of the disease. Even though the disease is not lethal, dogs with SA are sometimes euthanized because the treatment is life-long and labour-demanding. A clinically and cosmetically acceptable result is not always obtained.

No sex predilection has been reported for SA and onset of clinical signs are reported to appear in young adult to middle-aged dogs [13,14].

An autosomal recessive mode of inheritance with a variable expression has been proposed in the standard poodle [9,11] and an autosomal recessive mode is suggested for the akita [12].

The aim of this study was to investigate SA in dogs in Sweden with respect to breed, sex and age distribution. A second aim was to retrospectively compare clinical signs and estimate the survival after diagnosis in dogs with generalized SA in a group of dogs from the breeds springer spaniel (English springer spaniel), standard poodle and akita (Japanese akita).

## Methods

### Study design and collection of cases

Thirty-four Swedish veterinarians with recognized interest and knowledge in dermatology in 34 veterinary hospitals, clinics and centres throughout Sweden were contacted, in the text all denoted as "clinics". The yearly number of visits per clinic ranged from 3000 to 36,000. Efforts were made to ask the participating veterinarians to identify and contribute as many cases as possible irrespective of breed or severity of the disease, and no time-limit was given. The cases were located using mostly computerized clinical databases and through archives with histology reports at each clinic. All cases had to show clinical signs and also be confirmed by histopathology examination. Data on breed, sex and age at the time of diagnosis were registered from the clinical records.

### Breed distribution and comparison to registry data

The collected SA cases were placed in a ranking list according to the number of dogs per breed. In an attempt to estimate the relative occurrence, the number of dogs was compared breed-wise to official central registry data from the Swedish Board of Agriculture (SBA), in which all Swedish dogs should be registered (as of 31/10/2006).

The data (number of dogs per breed) were processed in the same way with central official registry data from the Swedish Kennel Club (SKK). The mean values for the yearly number of dogs that entered the SKK-registry during four years (2003–2006) were used as comparison. The two comparisons resulted in ranking lists based on relative occurrence, where ranking presumably was independent of the number of dogs of the breed in the country.

### Frequency comparison between three breeds in a clinic population

We included the SA cases from the springer spaniel, standard poodle and the akita from 11 of the clinics in the study during 3.5 years (01/01/2003–30/06/2006).

The number of SA cases in these clinics was compared to breed-specific attendance numbers from the same clinics during the same time period. Attendance numbers were based on the individual dogs and not on the number of visits. The Labrador retriever was selected as a "reference breed", being a common breed seldom diagnosed with SA.

Data consisted of clinical records from SA cases made available by the contact veterinarian in the clinic and attendance numbers made available by computer search in 10 clinics and manual counting in 1 clinic. These database searches were made by administrative personnel in 4 clinics and by the author in 7 clinics. Data regarding the reference breed was available from 6 clinics.

### Clinical signs, treatment and survival time in three breeds: the springer spaniel, standard poodle and the akita

Clinical signs at diagnosis and at follow-up, treatment, concurrent diseases and information about euthanasia were recorded for the springer spaniel, standard poodle and the akita by the use of a standardized questionnaire sent out to the attending veterinarians.

Dates for diagnosis and follow-up were registered. Extension of clinical signs was graded as localized or generalized, with localized defined as lesions only affecting either head, pinnae or ear canals. Dogs reported to only have localized clinical signs were excluded after this was determined. For each case of generalized SA the degree of alopecia, seborrhoea and overall severity of skin lesions were given a score from 1–3 (mild, moderate, severe). Number of events of pyoderma was graded as 1–3 (none, once, more than once). Presence of systemic clinical signs, pruritus, otitis externa and paw involvement was graded 0–1 (no, yes). Concurrent chronic disease was registered using free text.

Treatment protocols used were recorded as 0–1 (no, yes) for topical treatment, oral fatty acid supplements, oral corticosteroids and oral cyclosporine. Additional treatment was reported with free text. The last date for follow-up for each dog was recorded at the end of the study. Euthanasia was reported as no, yes or lost to follow-up. In case of euthanasia, the approximate date was registered and the reason for euthanasia was registered as SA, not SA or No Data.

### Statistical analysis

Fishers Exact Test was used to test for statistical significance with respect to the variables related to clinical signs from the questionnaire, comparing between breeds (springer spaniel, standard poodle and akita), age (1 < 3 years; 3 < 5 years; 5 < 6 years; > 6 years of age at diagnosis) and gender (male, female). If the p-value for the overall test was < 0.10, pair-wise comparisons were made and reported as significant when p < 0.05, the latter p-value was also used in the following analysis. McNemars test (for dichotomous variables) and Bowker's test of symmetry (> 2 categories) was used to assess the clinical signs at follow-up compared to at the time for diagnosis (in the 33 dogs with follow-up) with respect to breed, age and gender. The Kaplan-Meier test was used to determine the median survival time in springer spaniels, standard poodles and akitas combined, using dates for diagnosis and follow-up and information about euthanasia (alive, euthanized, euthanized due to SA) in dogs where this information was available. The SAS software was used for analysis (SAS Institute Inc., Cary, NC, 27513, USA).

## Results

### Swedish dogs with SA, an inventory

A total of 104 cases of SA were included in the study. Of the 34 veterinarians, 28 contributed cases to the study; 1 did not answer and 5 had no dogs with SA in their records. The cases were diagnosed during a period of 15 years (1992 to 2007), 91% during a period of 7 years (2000 to 2007). The 104 cases were distributed among 25 breeds (mixed breeds excluded). Most cases were recorded for the springer spaniel (n = 25), standard poodle (n = 21) and the akita (n = 10). The breed distribution is presented in Table 1.

**Table 1:** Breed distribution in the study of 104 dogs with sebaceous adenitis

Breeds	No. of Dogs	Breeds, continued	No. of Dogs	Breeds, continued	No. of Dogs
springer spaniel	25	standard schnauzer	2	Newfoundland	1
standard poodle	21	eurasier	2	Old English sheepdog	1
akita	10	St Bernhard	2	Chinese crested dog	1
lhasa apso	6	havanese	1	rottweiler	1
chow-chow	3	boxer	1	samoyed	1
flatcoated retriever	3	briard	1	vizla	1
Bernese mountain dog	2	collie	1	Welsh springer spaniel	1
hovawart	2	coton de tuléar	1	mixed breeds	11
Labrador retriever	2	golden retriever	1	Total	104

### Comparison to registry data

The results from the comparisons to registry data are shown in Table 2. The relative listing of data resulted in similar ranking lists where the same breeds constituted the "top-five" in both comparisons. These were the akita, standard poodle, lhasa apso, chow-chow and the springer spaniel. The akita showed substantively higher relative occurrence compared to the other breeds.

**Table 2:** The dogs in the study (n = 104) – comparison to registry data.

Breeds^1^	Number of dogs	Number in SBA-registry	Proportion relative to SBA	Ranking
akita	10	195	0.051	1
standard poodle	21	3439	0.006	2
lhasa apso	6	1062	0.006	3
chow-chow	3	688	0.004	4
springer spaniel	25	6308	0.004	5
flatcoated retriever	3	5837	0.0005	6
Breeds^1^	Number of dogs	Number of new registrations in SKK-registry	Proportion relative to SKK	Ranking
akita	10	24	0.417	1
lhasa apso	6	166	0.036	2
standard poodle	21	651	0.032	3
chow-chow	3	103	0.029	4
springer spaniel	25	1139	0.022	5
flatcoated retriever	3	1188	0.003	6

^1 ^= The six numerically most common breeds among the dogs in the study (n ≥ 3)

### Age at diagnosis and gender

The mean age at diagnosis was 4.8 years (median 5 years; n = 103) ranging from 1 to 11 years, with 40% diagnosed at the age of ≥6 years. For the springer spaniel (n = 25), the mean age at diagnosis was 4.5 years; for the standard poodle (n = 21), 5 years and for the akita (n = 10), 4.9 years. The proportion of males were 61% in the entire group; for the springer spaniel 68%, for the standard poodle 62% and for the akita 40%.

### A frequency comparison in population from 11 clinics

The population of dogs from the 11 clinics studied consisted of springer spaniel (n = 2341), standard poodle (n = 1367), akita (n = 149) and Labrador retriever (n = 4396). SA was recorded in 13 springer spaniels (0.6%), 12 standard poodles (0.9%) and 5 akitas (3.4%). No Labrador retrievers were diagnosed with SA.

### Clinical signs, treatment and median survival time in three breeds: the springer spaniel, standard poodle and the akita

All springer spaniels, standard poodles and akitas diagnosed with SA (n = 56) were selected for the survey. The attending veterinarians (n = 21) assisted with information (100% response rate). Dogs reported only showing localized clinical signs (n = 5) were excluded. Clinical signs, treatment protocol and follow-up were compiled for the remaining 51 dogs.

### Clinical signs at diagnosis

The breed distribution was as follows: springer spaniel (n = 24), standard poodle (n = 19) and akita (n = 8). The number of dogs with reported degrees of clinical signs is presented in Table 3. The springer spaniel had consistent and significantly higher clinical scores (more severe signs) compared to the standard poodle for several variables. For alopecia (graded mild, moderate, severe) the percentages for the springer spaniel were 25%, 46%, 29% compared to the poodle 47%, 53%, 0% (p = 0.02). With respect to seborrhoea the percentages were 13%, 33%, 54% for the spaniel compared to the poodle 26%, 58%, 16% (p = 0.04). Overall severity of clinical signs (mild to severe) in the spaniel were 8%, 46%, 46% compared to the poodle 21%, 68%, 11% (p = 0.03). Furthermore, events of pyoderma (graded none, once, more than once) were 21%, 37%, 42% for the springer spaniel and 63%, 16%, 21% for the poodle (p = 0.02). Moreover the akita exhibited more severe clinical signs as compared to the poodle, although not statistically significant for most variables. However, for pyoderma the percentages in the akita were 25%, 0%, 75% (p = 0.05).

**Table 3:** Degree of clinical signs in 51 dogs with SA at diagnosis and in 33 dogs at follow-up.

	Category	springer spaniel	standard poodle	akita	total
At diagnosis	Number of dogs	24	19	8	51
	Graded as mild = 1, moderate = 2, severe = 3 in number of dogs				
	Alopecia at diagnosis	6, 11, 7	9, 10, 0	2, 5, 1	17, 26, 8
	Seborrhoea at diagnosis	3, 8, 13	5, 11, 3	0, 6, 2	8, 25, 18
	Pyoderma up to diagnosis ^1^	5, 9, 10	12, 3, 4	2, 0, 6	19, 12, 20
	Severity of signs at diagnosis	2, 11, 11	4, 13, 2	2, 3, 3	8, 27, 16
	Graded as no = 0, yes = 1 in number of dogs				
	Pruritus at diagnosis	10, 14	11, 8	2, 6	23, 28
	Otitis up to diagnosis	14, 10	14, 5	7, 1	35, 16
	Paw involvement at diagnosis	20, 4	16, 3	5, 3	41, 10
	General signs/discomfort	21, 3	14, 5	5, 3	40, 11
At follow-up	Number of dogs	14	14	5	33
	Graded as mild = 1, moderate = 2, severe = 3 in number of dogs				
	Alopecia at follow-up	9, 5, 0	7, 5, 2	1, 2, 2	17, 12, 4
	Seborrhoea at follow-up	7, 3, 4	5, 6, 3	1, 1, 3	13, 10, 10
	Pyoderma during follow-up^1^	3, 1, 10	10, 2, 2	0, 1, 4	13, 4, 16
	Severity of signs at follow-up	7, 3, 4	8, 1, 5	1, 2, 2	16, 6, 11
	Graded as no = 0, yes = 1 in number of dogs				
	Pruritus at follow-up	8, 6	10, 4	3, 2	21, 12
	Otitis during follow-up	6, 8	11, 3	5, 0	22, 11
	Paw involvement at follow-up	11, 3	12, 2	2, 3	25, 8

^1 ^= the grading refers to "events of pyoderma", 1 = none, 2 = once, 3 = more than once.

No significances were found regarding the different variables (clinical signs) compared to age or gender, except from the reported impairment of clinical attitude associated with SA. Of the 11 dogs reported to show general signs with the disease, 9 were younger than 5 years (p = 0.03).

Concurrent chronic disease was reported in 22 dogs (43%). The most common diseases were hypothyroidism (n = 8) and atopy (n = 4). Among other reported chronic diseases were hypo- and hyperadrenocorticism, different tumours, urolithisasis, lupoid onychodystrophy and epilepsy.

### Treatment, follow-up and survival

Of all the dogs (n = 51), topical treatment had been prescribed in 98%, oral FA in 96%, oral corticosteroids in 55% and oral cyclosporine in 25%. Additional treatment was recommended in 16% of the dogs, in all dogs the additional treatment referred to vitamin A.

Follow-up time was 12 months or longer for 33 of the 51 dogs. Mean follow-up time for the 33 dogs were 34 months (median = 21 months) and 1 dog was regularly seen for 108 months.

In the paired comparisons of clinical scores at diagnosis and at follow-up (n = 33), significant improvement was seen regarding seborrhoea and the degree of severity. For seborrhoea (graded mild, moderate, severe) the clinical scores at diagnosis were 15%, 55%, 30% and improved to at follow-up 39%, 30%, 30% (p = 0.04). For the estimated degree of severity (mild, moderate, severe) the figures at diagnosis were 15%, 55%, 30% and at follow-up 48%, 18%, 33% (p = 0.01).

At follow-up breeds differed significantly with respect to two variables. For pyoderma (graded none, once, more than once) the recorded figures were for springer spaniel (n = 14) 21%, 7%, 71%; for poodle (n = 14), 71%, 14%, 14% and for akita (n = 5), 0%, 20%, 80% (p = 0.002). Moreover, events of otitis externa differed significantly during follow-up (p = 0.04) with 57% of the springer spaniels affected as compared to 21% of the poodles and none of the akitas.

At the end of the study, information with respect to survival was recorded in 44 dogs, median survival time was 42 months (95% CI 27–108), whereas in 7 dogs this information was not available. Furthermore, after the median survival was reached, the point estimate of the survival remained the same until month 108. Euthanasia due to SA was reported in 14 of the 44 dogs where information about survival was available, whereof 7 (all springer spaniels or akitas) were euthanized within 2 years after diagnosis. Three years after diagnosis 11 dogs had been euthanized due to the disease. Of the euthanized dogs, 11 were younger than 10 years at the time.

## Discussion

Because SA is an unusual disease, the retrospective study-design could be justified. Effort was made to collect as many cases as possible. In this study, cases were included if they had clinical signs and histopathology compatible with what is described for SA in the literature. The sebaceous glands can be involved and destroyed even in inflammatory reactions not primarily targeting the glands. This could partly explain both the inconsistent clinical signs sometimes seen in dogs with SA [19] and the high number of different breeds described with the disease. Furunculosis and demodicosis severe enough to involve the sebaceous gland can mimic SA, which is the case in feline acne [20]. SA has also been described with thymoma and other serious internal disease in the rabbit [4,21,22] and in dogs with leishmaniosis [19,23]. In our material it is not ruled out that the inflammatory attack of the sebaceous glands could have different ethiologies. This is why we excluded breeds with few cases and cases with only local signs from further investigation in the study.

### Breeds

SA proved to be an uncommon disease in this material, which is in agreement with what has earlier been reported [13]. The highest number of cases was found in the springer spaniel breed, followed by the standard poodle and the akita. When comparing to the different population or clinic data, the springer spaniel still belonged to the five most frequently diagnosed breeds, although not longer highest ranked. To our knowledge, overrepresentation of SA in the springer spaniel has not previously been reported. Interestingly, the lhasa apso and the chow-chow also belonged to the five most common breeds in the ranking list but were not further studied.

The results were also compared to data made available by the Swedish pathology laboratory BioVet AB, consisting of 229 dogs, histologically diagnosed with SA at the laboratory during the years 1996 to 2005. The breed distribution is presented in Table 4. When the numbers per breed in the report (breeds with ≥6 dogs) were ranked with the same method as for the cases in the study, the ranking list constituted of, using a descending scale, the akita, chow-chow, lhasa apso, springer spaniel and the standard poodle, which are the same breeds as in the results for the study. Regarding the 229 dogs in the report further information other than histopathological diagnosis and breed was not available. Thus the material is used only for comparison.

**Table 4:** Breeds diagnosed with sebaceous adenitis at the pathology laboratory BioVet AB year 1996 to 2005

Breeds ^1^	No. of dogs	Breeds, continued	No. of dogs
English springer spaniel	30	border collie	4
akita	16	Shetland sheepdog	4
standard poodle	15	jack russel terrier	4
Bernese mountain dog	9	beagle	3
German shepherd dog	8	Welsh springer spaniel	3
lhasa apso	8	briard	3
Labrador Retriever	7	rottweiler	3
chow-chow	6	standard schnauzer	3
samoyed	4	bichon frisé	3
flatcoated retriever	4	dachshound, wirecoated	2

^1 ^= Another 49 breeds were presented in the report, with 1–2 dogs per breed.

SA is well known to breeders and owners of standard poodles. This may result in dogs not becoming diagnosed with histopathology and therefore give the impression that SA is less common than it is. However, it is also possible that many poodles get diagnosed because the breed association encourages poodle owners to test their dogs for SA, by the use of biopsies for histopathology examination, a request before mating. The number of akita in Sweden is small and the occurrence of SA is high compared to the other breeds. On the other hand, the English springer spaniel is a common breed, and our impression is that owners and breeders are not at all familiar with the disease.

### Age

The variable "age at diagnosis" was recorded because this information is one of the indisputable facts in a retrospective study. SA is reported in the literature to occur in young adult to middle-aged dogs [13,14], referring to the onset of clinical signs. The time period between the onset of clinical signs and the date of verified diagnosis is highly likely to be variable. Given this, it is still worth noting that 40% of all the dogs were ≥6 years at time for diagnosis; for springer spaniel 28%, poodle 43% and akita 50%. This should be compared with the results from Reichler (2001) [12], where 30% of the 23 akitas in a study were older than 5 years at first recognized signs and those from Dunstan (1995) where 10% of the standard poodles were > 5 years at onset [11].

### Gender

Males were overrepresented in the entire group, as well as in the standard poodles and the springer spaniel. There is no apparent reason why a predisposition was seen for males. The result differs from previous studies where no sex predilection have been reported [13,14]. Except from German Shepherd dog pyoderma, where a predominance for males was shown by Koch & Peters (1996) and by Denerolle et al (1998) [24,25], no sex predilection is seen for deep pyoderma in dogs at the authors knowledge, which could otherwise be a cause of secondary inflammation and destruction, at least clinically, of the sebaceous glands.

### Clinical signs

The results implicates that the springer spaniels have more severe clinical signs of SA than the standard poodle, to our knowledge not previously described. This study found that akita can have severe signs of the disease, which agrees with findings of Reichler [12].

Pruritus was reported in 55% of all dogs (28/51 dogs) and 63% of the dogs (32/51 dogs) had been diagnosed with pyoderma at least once prior to diagnosis. However, also 47% of the dogs (9/19 dogs) reported to not have had pyoderma were pruritic. This indicates that SA can be a primarily pruritic condition or that pruritus could be due to skin infections, ectoparasites or allergic conditions not diagnosed by the attending veterinarian. Otitis externa was commonly reported in the springer spaniel (10/24 dogs) compared to the akita (1/8 dogs). The ear diseases were not investigated or categorized further and because otitis is common in all Spaniels it could be explained by anatomical differences as well as by concurrent disease, for example atopy.

It has been suggested that the akita can show general signs or fever associated with the disease [1,10,13] but this could not be confirmed by Reichler in 2001 [12]. In the present study an impaired general attitude was reported in 22% of the dogs, not significantly different among the three breeds but significantly more common in the younger dogs. This depression in attitude was described especially in some dogs with intermittent clinical signs (data not shown), a finding also described by Rosser 1992 [10].

As could be seen in the results (Table 4) the options for many questions were mild/moderate/severe. In a few occasions (9 of 336 observations), the responsible veterinarian wrote beside the boxes or marked two boxes. The observation "none" in free text beside the box for mild in a question were processed statistically as mild and when two boxes were marked, the answer was recorded as the lower of the two. The reported results are therefore possibly somewhat more severe compared to if the questionnaires contained the option "none" as an alternative in the questions.

### Concurrent diseases

Eight dogs were reported to be hypothyroid. Hypothyroidism is common in middle-aged dogs and the disease can be both under- and over-diagnosed. Some of the dogs in the study may have been euthyroid because only a total T4 level below normal value was recorded before institution of thyroid supplementation. All four atopic dogs were either akita or springer spaniels: however, because atopy is a common disease [13] it is not possible to draw further conclusion from this finding.

### Treatment, follow-up and outcome

At follow-up, the degree of clinical signs varied; some variables improved significantly and others did not. The springer spaniel and the akita exhibited more severe clinical scores, especially with respect to pyoderma, as compared to the standard poodle.

The fact that at least 14 of the 51 dogs, from the investigated three breeds, were euthanized, mainly due to the disease, demonstrates that SA can be a challenging disease, sometimes with a poor prognosis. The reason for the poor prognosis might be that a full recovery is often not obtained and the treatment protocol is labour-demanding for the owner. A high proportion of the dogs were only recommended symptomatic treatment with topical care and oral fatty acid supplementation. This may indicate that many of the dogs had mild clinical signs. However, it may also indicate that by adding oral treatment, the patients might have been more successfully managed with less need for time-consuming topical treatment. We therefore hypothesise that systemic treatment may have prolonged the survival in the studied dogs. Only springer spaniels and akitas (n = 7) were reported euthanized within 24 months after diagnosis, possibly indicating a more severe phenotype of the disease in these breeds. In seven dogs (6 springer spaniels, 1 poodle) no information regarding survival was available at the end of the study.

### Comparison with SBA and SKK

The central register at the SBA is compulsory for all dogs in Sweden. However, some dogs likely never get registered and deceased dogs are sometimes not removed from the active register. Nevertheless, there is no reason to believe that these biases would be more pronounced in one breed over another. Therefore the register was used for the purpose of comparison needed in this study.

The official register run by the SKK is a register where new dogs, puppies and imports are registered. We used the number of dogs of the different breeds that entered the SKK-registry each year during four years, as a measurement of how common the certain breeds in the study were in Sweden at the time. There is presumably a lack in registration also in this register, but probably not substantially differential with regard to breed. Together, especially as the comparisons showed similar results, the methods were considered satisfactory for the purpose of presenting a ranking list with some relevance to the relative occurrence within breeds.

### Search motors and databases

One challenge was to identify the SA cases in different clinical recording systems used in the 11 clinics. Reasons for not finding all SA dogs could be several. Some SA dogs may have visited the clinic due to other reasons than SA and therefore not be diagnosed, or more importantly they could have been diagnosed with SA, for example after biopsy, without having the diagnosis updated in the clinical record. Further, some of the diagnostic registries had no SA code. It was therefore deemed necessary to scrutinise cases diagnosed with for example seborrhoea, alopecia and immune-related skin diseases. Theoretically, built-in functions for up-dating of diagnoses after confirmation could have made case finding more complete. Moreover, better built-in search facilities, for example for searching free text, could have contributed in the case-finding process. Based on our experiences, we conclude that only a few of the commercial computerized record systems in Sweden have sufficient case-finding functions for undertaking effective retrospective clinical follow-up or research, at least with respect to SA and from multiple clinics.

## Conclusion

In the present study of 104 dogs with SA, the disease was most often seen in the akita, standard poodle, lhasa apso, chow-chow and the springer spaniel. The results suggest that the springer spaniel belongs in the group of predisposed breeds. When the springer spaniels, standard poodles and the akitas in the study were compared, the springer spaniel showed significantly more severe clinical signs compared to the standard poodle with respect to alopecia, seborrhoea, pyoderma and the overall severity of clinical signs. A large proportion of dogs were euthanized mainly because of the disease. Only springer spaniels and akitas were reported euthanized due to the disease within 24 months after diagnosis. More studies are needed for further exploration of the disease and the inheritance mode in the springer spaniel.

## Competing interests

The authors declare that they have no competing interests.

## Authors' contributions

EHT designed and carried out the study, collected the data, drafted and revised the manuscript, KB contributed to the study with important scientific advice and with critical revising of the manuscript, AE contributed substantially to the statistics work and supported the writing process. All authors read and approved the final manuscript.
